# 

**Published:** 1878-12

**Authors:** 


### CONTENTS:

ARTICLE I.—Maryland and District of Columbia Dental Association. (Continued.)... 337

ARTICLE II.—"Plastic Filling" as a "Power" in Dentistry. By J. Foster Flagg, D. D. S.... 347

ARTICLE III.—Nœvus, or Effects of Dental Operations during the Period of Utero-Gestation.... 358

ARTICLE IV.—"Bleaching Teeth." By J. Taft, D. D. S.... 364

ARTICLE V.—Chloramyl, a new Anæsthetic and an Improved Inhaler. By George E. Sanford, M. D.... 371

ARTICLE VI.—Loss of Blood in Surgical Operations. By Alfred Fuchs, D. D. S.... 374

### EDITORIAL, ETC.

Anæsthetics for Children.... 376

### OBITUARY.

The Late Dr. Philip H. Austen.... 377

### BIBLIOGRAPHICAL.

The Advantages and Accidents of Artificial Anæsthesia.... 379

Treatment of the Genito-Urinary Organs.... 380

The Scientific American.... 381

The American Agriculturist.... 382

Manual of Medical Electricity.... 383

Virginia Medical Monthly.... 383

New Electrodes and Battery for Electrolysis of Uterine Tumors.... 383

### MONTHLY SUMMARY.

Turpentine Vapor in Accidents from Chloroform.... 384

A Case of Third Dentition.... 384

